# 3D high-resolution atrial wall thickness maps using black-blood PSIR

**DOI:** 10.1186/1532-429X-17-S1-P239

**Published:** 2015-02-03

**Authors:** Marta Varela, Christoph Kolbitsch, Adeline Theron, Ross Morgan, Markus Henningsson, Tobias Schaeffter, Oleg Aslanidi

**Affiliations:** Biomedical Engineering, King’s College London, Kragujevac, UK

## Background

Atrial fibrillation (AF) is the most common cardiac arrhythmia. It is often treated using catheter ablation, which aims to isolate arrhythmogenic regions by delivering localized energy. Whereas an insufficient energy delivery can lead to AF recurrence, excessive power can cause potentially lethal complications. Knowledge of atrial wall thickness can help select an optimal amount of energy, but clinical imaging does not currently provide atrial wall thickness data for patients. Even ex-vivo studies have only measured the thickness of the atrial wall in discrete locations using CT or post-mortem samples.

In this study, we apply black-blood MR imaging to reconstruct both epi- and endocardial surfaces of the entire atria and compute wall thickness maps in healthy volunteers. To our knowledge, this is the first study to provide a complete 3D map of the wall thickness of both right and left atria.

## Methods

4 healthy volunteers (3 male, 21-31 years old) were successfully imaged with ethical approval using a phase-sensitive inversion recovery sequence (PSIR). All data was acquired on a Philips 3T Achieva, with 3D FLASH, typical FOV: 280 x 190 x 120 mm^3^, 1.4-mm isotropic resolution, FA: 20°, TE/TR/TI: 2.7/5.9/120 ms; respiratory gating using a pencil-beam navigator; average scan time: 12 min. Cardiac triggering was used to ensure data acquisition was carried out in mid atrial diastole.

Manual segmentation of the epi- and endocardial surfaces from the images was performed using ITK-SNAP. Triangular meshes of each of the surfaces were generated using Matlab and smoothed with a curvature flow operator (mean internode distance: 0.56 mm). Wall thickness was then computed by: A) measuring the distance between each node in the endocardial surface and its nearest neighbour in the epicardial surface; B) repeating procedure A in reverse for each node in the epicardial wall and C) averaging the outcomes of procedures A and B.

## Results

**Figure 1:**
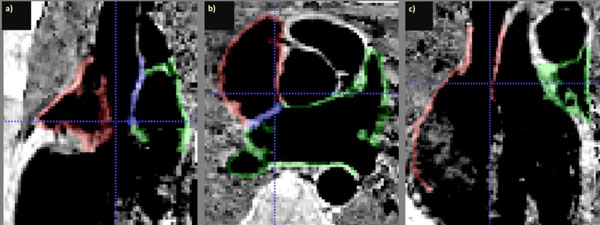
a) Sagittal, b) transverse and c) coronal images of the atria, overlaid with the atrial wall segmentation. Red: right atrial wall, green: left atrial wall; blue: septum.

**Figure 2:**
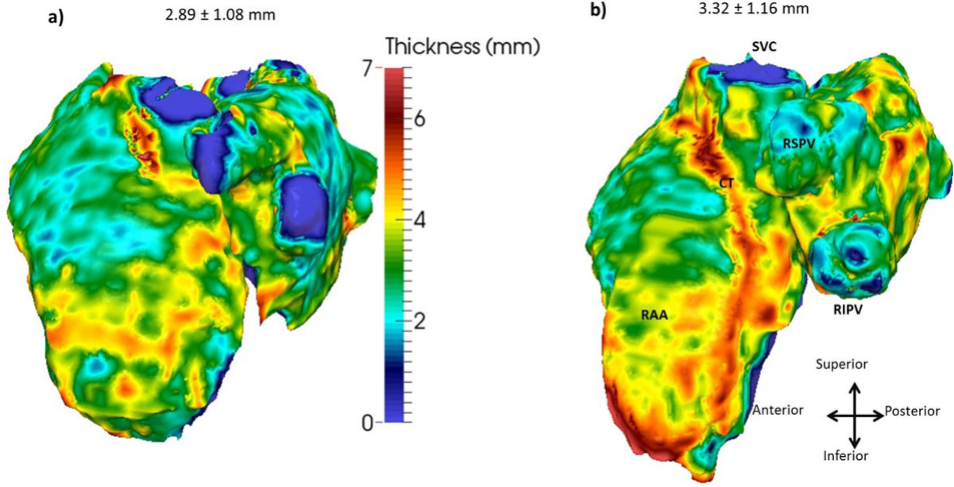
Atrial wall thickness maps in two representative subjects (mean ± standard deviation in each). CT: crista terminalis; RAA: right atrial appendage; SVC: superior vena cava and right superior/inferior pulmonary veins (RSPV/RIPV).

Blood signal was strongly attenuated in relation to the myocardium, allowing the reconstruction of both the epi- and endocardial surfaces from the PSIR images (Fig. 1). The measured atrial wall thickness for each subject is mapped onto the epicardial surface and shown in Fig. 2. Wall thickness was on average 3.01 ± 1.09 mm (range: 2.80 ± 1.05 to 3.32 ± 1.16 mm), in good agreement with post-mortem data. The distinctive atrial bundle of the crista terminalis is clearly visible as a ridge with increased thickness and the pulmonary vein sleeves are thinner than other regions of the atria.

## Conclusions

We developed a protocol that allows us to obtain 3D high-resolution bi-atrial wall thickness maps in healthy volunteers. Additional data is being acquired to create an atrial wall thickness atlas of healthy subjects. Future work includes acquiring data from AF patients to quantify the atrial wall changes caused by disease and provide a tool to aid catheter ablation treatments.

## Funding

British Heart Foundation and KCL Centre of Excellence in Medical Engineering.

